# Polyphenol dietary supplementation prevents inflammation‐induced muscle atrophy in a zebrafish ( *Danio rerio* ) model

**DOI:** 10.1002/jsfa.70932

**Published:** 2026-07-25

**Authors:** Graziella Orso, Monica Matuozzo, Mariapina Rocco, Seyed Hossein Hoseinifar, Ghasem Ashouri, Andrea Scaloni, Marina Paolucci, Chiara D'Ambrosio, Roberta Imperatore

**Affiliations:** ^1^ Department of Sciences and Technologies University of Sannio Benevento Italy; ^2^ Proteomics, Metabolomics and Mass Spectrometry Laboratory, ISPAAM‐National Research Council Portici Italy; ^3^ Department of Fisheries Gorgan University of Agricultural Sciences and Natural Resources Gorgan Iran

**Keywords:** muscle degeneration, chestnut tannins, apigenin, functional food, NF‐*κ*B signaling, proteomics

## Abstract

**BACKGROUND:**

Diet‐induced inflammation is a major cause of muscle degeneration impacting human and animal health. Owing to their anti‐inflammatory and antioxidant properties, polyphenols have attracted considerable interest as feed additives. Using a combined immunohistochemical, western blotting (WB), and proteomic approach, we tested the hypothesis that a pro‐inflammatory diet in zebrafish (*Danio rerio*) induces skeletal muscle degeneration, and that dietary polyphenols can prevent or reverse this effect. Fish were supplemented with a blend of polyphenol extracts, tannins from *Castanea sativa* shells and apigenin from *Verbascum macrurum*, administered before (PVpreI), during (PVwithI), or after (PVpostI) the inflammatory diet.

**RESULTS:**

Inflammation led to muscle fiber degeneration, expanded intercellular space, and increased TNF‐*α*, COX2, and caspase 3 expression. Polyphenol supplementation prevented or attenuated these alterations and reduced expression of inflammatory markers. WB revealed changes in I*κ*B*α* phosphorylation, desmin, and titin levels in inflamed muscle, fully or partially restored in PVpreI and PVwithI groups. Proteomics of inflamed muscles showed quantitative alterations in structural and regeneration‐related proteins, connective tissue components, inflammatory and immune mediators, and metabolite transporters, reflecting the observed morphological and molecular changes, which were in most cases reverted in polyphenol‐treated groups.

**CONCLUSION:**

These outcomes demonstrate that dietary supplementation with a specific polyphenol blend counteracts muscle atrophy by modulating the proteomic landscape. Among treatments, polyphenol pre‐treatment was the most effective in preventing inflammation‐induced damage, followed by co‐ and post‐treatment, which showed partial recovery. These findings strongly support the use of dietary polyphenols as a robust nutritional strategy to preserve muscle integrity during systemic inflammation. © 2026 The Author(s). *Journal of the Science of Food and Agriculture* published by John Wiley & Sons Ltd on behalf of Society of Chemical Industry.

## INTRODUCTION

A growing body of evidence suggests that diets with pro‐inflammatory properties, such as diets rich in ultra‐processed foods and low in fruits and vegetables and/or high‐fat diet, may induce a low‐grade systemic inflammation^1^, ^2^, ^3^ which is key to skeletal muscle loss or atrophy.^4^ Some of the cellular processes that contribute to this are: (i) release of pro‐inflammatory cytokines like interleukin‐1*β*, interleukin‐6, and tumor necrosis factor‐*α* (TNF‐*α*), which inhibit synthesis and accelerate degradation of proteins by modulating signaling pathways such as JAK/STAT3, ERK, and PI3K/Akt; (ii) activation of protein breakdown via calpains, ubiquitin–proteasome system, and caspase 3; (iii) enhanced lysosomal autophagy through p38/MAPK pathway activation; (iv) increased TGF‐*β* activity; and (v) mTOR pathway downregulation.^5^, ^6^ In skeletal muscle, inflammation triggers reactive oxygen species production, apoptosis, mitochondrial dysfunction, and reduced antioxidant defenses.^7^, ^8^, ^9^ These mechanisms disrupt the balance between protein synthesis and degradation, leading to atrophy.^10^ Inflamed muscle in mice shows upregulation of embryonic myosin forms, altered actin–myosin interactions, and reduced contractility.^11^, ^12^ Desmin, another muscle‐related protein, is upregulated during atrophy.^12^

The maintenance of muscle integrity is critical for both human clinical outcomes and the sustainability of animal production systems.^10^, ^11^, ^12^, ^13^ Due to the critical link between diet, inflammation, and muscle health, research focuses on optimizing human and animal nutrition.^14^ A promising approach is using natural compounds that regulate the balance of pro‐/anti‐inflammatory factors and muscle protein turnover.^15^, ^16^ Polyphenols are especially relevant for their anti‐inflammatory and antioxidant properties.^17^ Lipophilic curcumin, for example, counteracted muscle atrophy in a mouse model of LPS‐induced inflammation by inhibiting the p38/MAPK pathway, reducing inflammation and oxidative stress.^18^ Resveratrol alleviated muscle atrophy in mouse models of diabetes, cancer, and denervation by inhibiting NF‐*κ*B.^19^, ^20^ Quercetin dietary supplementation prevented muscle inflammation and atrophy caused by high‐fat diets in mice by downregulating TNF‐*α*.^21^ Although the positive effects of certain polyphenols on inflammation and oxidative status are documented, their specific impact on diet‐induced muscle atrophy remains underexplored.

Zebrafish (*Danio rerio*) have become a valuable translational model for studying inflammation, serving as both a representative species for aquaculture research and a robust vertebrate model for biomedical studies.^22^, ^23^, ^24^ Their use also aligns with the EU ‘Three Rs’ guidelines: replacement, reduction, and refinement.^25^ Inflammation can be easily triggered in zebrafish by using biological or mechanical sources such as inflammatory diet or injury, respectively, thus allowing rapid screening of anti‐inflammatory therapies, as the primary pro‐ and anti‐inflammatory cytokines are highly conserved between zebrafish and mammals.^25^

Building upon our previous findings that chestnut shell and *Verbascum* polyphenol extracts can restore intestinal homeostasis in inflamed zebrafish,^26^ the study presented here aimed to determine if the localized inflammation and polyphenol beneficial effects extend to peripheral tissues. Specifically, we investigated whether counteracting diet‐induced intestinal inflammation can subsequently arrest the systemic cascade that leads to skeletal muscle wasting. Given the limited knowledge about polyphenols' role in diet‐induced muscle atrophy, we used a multiscale approach combining immunohistochemistry, western blotting (WB), and high‐resolution proteomics to test whether a pro‐inflammatory diet induces muscle atrophy and if a polyphenol‐enriched diet can reverse it governing muscle protein turnover. This approach has successfully identified physiologically relevant molecules and biomarkers in zebrafish.^27^

## MATERIALS AND METHODS

### Phytochemical profiling and chromatographic data re‐evaluation

The polyphenols were extracted from chestnut (*Castanea sativa*) shell coming from Montella (a geographical area in the province of Avellino, Italy) and *Verbascum macrurum* harvested in Pietraroia (an area in the province of Benevento, Italy).^26^ To provide accurate quantitative data on the bioactive components of the tested extracts, a retrospective quantitative analysis was performed on the original high‐performance liquid chromatography (HPLC) with diode array detection datasets obtained during the 2022 analytical sessions.^26^, ^28^ The original analyses were conducted using methanolic extracts of *V. macrurum* at a concentration of 0.5 mg mL^−1^ and aqueous extracts of *C. sativa* shell at 50 mg mL^−1^, as previously described for qualitative compound profiling.^26^ Prior to quantification, raw chromatographic profiles were subjected to advanced digital preprocessing using OriginPro software (OriginLab Corporation, Northampton, MA, USA) to ensure baseline stability, remove background noise, and optimize peak integration. Due to the different chemical nature and complexity of the two plant matrices, two distinct quantification strategies were adopted. Specifically, (i) *V. macrurum* extract: individual major bioactive compounds, namely quercetin and ellagic acid, were quantified using their respective external standard calibration curves. Apigenin was quantified at 280 nm as luteolin equivalent (LE) using a standard calibration curve of luteolin, selected as a representative standard for the flavone chemical class. Total polyphenol content was calculated via HPLC as the sum of all peak areas recorded at 280 nm and expressed as milligrams of gallic acid equivalents per gram of dry extract (mg GAE g^−1^). (ii) *C. sativa* shell extract: given the highly complex, oligomeric and polymeric nature of the chestnut matrix, a class‐specific quantification approach was applied by extracting the preprocessed chromatograms at specific characteristic wavelengths. Total polyphenol content was calculated at 275 nm as the sum of peak areas and expressed as mg GAE g^−1^. Hydroxybenzoic acids were quantified from the chromatograms extracted at 260 nm and expressed as syringic acid equivalents (mg SAE g^−1^); hydroxycinnamic acids were quantified at 325 nm and expressed as ferulic acid equivalents (mg FAE g^−1^); flavonols were monitored at 375 nm as kaempferol equivalents (KE) but results were below the detection limit. Gallic acid, individually resolved in the chromatogram, was quantified using its own reference standard calibration curve. Finally, the total tannin content (including both hydrolyzable and condensed forms) was estimated through a mass‐balance subtraction method, by subtracting the individual quantified contributions of hydroxycinnamic acids, hydroxybenzoic acids, and flavonols from the total phenolic content measured by HPLC monitoring at 275 nm.

### Zebrafish rearing and experimental diets

The zebrafish muscle samples analyzed in this work were harvested from the experimental trial described by Imperatore *et al*.^26^; thus, the experimental conditions will be only briefly reported here. While that previous work focused on intestinal morphology and microbiota, the current study utilized entirely distinct samples and specialized techniques (proteomics and WB) to address a separate biological question regarding muscle‐specific damages. Male adult zebrafish (*Danio rerio*), about 400 ± 100 mg (mean ± SD), were bred at the Vakili ornamental fish production complex, with the permission of the Department of Fisheries, Gorgan University of Agricultural Sciences and Natural Resources (GUASNR), Gorgan, Iran. The GUASNR guidelines for animal husbandry were followed during the animal experiments. Measurements were taken to reduce fish suffering. Before or during the experimentation, no medical treatment was provided. No deaths occurred.

Animals were fed once daily at 20 g kg^−1^ of body weight. Zebrafish were divided into six dietary groups (*n* = 36 zebrafish per group; *n* = 12 fish per tank). (i) Animals fed once a day with peeled *Artemia salina* cysts (220 g kg^−1^ fat, 440 g kg^−1^ proteins, 160 g kg^−1^ carbohydrates; Aqua Schwarz); control group (CT). (ii) Animals fed once a day for 10 days with *A. salina* supplemented with 1 g kg^−1^ k‐carrageenan; inflamed group (I). (iii) Animals fed once a day for 10 days with *A. salina* added with polyphenol extracts followed by 10 days of inflammatory diet; polyphenols pre‐treated inflamed group (PVpreI). (iv) Animals fed once a day for 10 days with inflammatory diet and polyphenol extracts; polyphenols co‐treated inflamed group (PVwithI). (v) Animals fed once a day for 10 days with inflammatory diet followed by *A. salina* added with polyphenol extracts for 10 days; polyphenols post‐treated inflamed group (PVpostI). (vi) Animals fed once a day for 10 days with *A. salina* added with polyphenol extracts; polyphenol‐treated control group (PV).^26^ The dietary concentration of 1 g kg^−1^ k‐carrageenan was selected based on our previous studies validating its effectiveness in establishing a robust, nonlethal model of systemic and localized inflammation in adult zebrafish.^26^, ^28^ The dietary inclusion of this blend was formulated so that each fish received a standardized daily intake of 4 μg per fish of chestnut shell extract and 2 μg per fish of *V. macrurum* extract. This precise dosage was determined based on prior dose‐finding work,^28^ implemented in a subsequent multi‐extract feeding trial,^26^ and corresponds to the adjusted average bioactive intake observed in reference diet models.^29^ To ensure absolute chemical consistency and reproducibility, the current study utilized the same standardized batch of extracts thoroughly profiled in our previous works.^26^, ^28^ After the feeding trial, zebrafish were fasted overnight and sacrificed (*n* = 18 fish per group; *n* = 6 fish per tank). The skeletal muscle tissue for WB and proteomics analysis was rapidly dissected on ice, frozen in liquid nitrogen, and stored at −80 °C until use.

### Histological and immunohistochemical analysis

Histological and immunohistochemical analyses were performed using the method described in previous work.^26^ Six adult zebrafish muscle samples were collected for histological analysis from each group, fixed in 4% (w/v) formalin, dehydrated in alcohol, clarified in xylene, embedded in paraffin, sliced into 5 μm sections, and stained with hematoxylin–eosin or with primary antibodies anti‐TNF‐*α* (1:200; NBP1‐19532, Novus Biologicals, USA), COX2 (1:200; 160126, Cayman), and caspase 3 (1:200; code ab13847, Abcam, Cambridge, UK). A light microscope (Leica DMI6000) equipped with a digital CCD camera (Leica DFC340, Leica Microsystems) was used to capture microscopic images (×20 and ×40 magnifications) and analyze morphological and immunohistological changes in skeletal muscle tissues. Micrographs were adjusted for light and contrast using Adobe Photoshop 6.01 (Adobe Systems, San Jose, CA) and then assembled on plates. The number of caspase 3‐positive cells was defined following a method previously reported.^29^ To measure the average muscle fiber cross‐sectional area (CSA), three randomly selected images per muscle from three representative fields of tissue for each animal per experimental condition (*n* = 6 animals per group) were analyzed using ImageJ software Version 1.52n, which was equipped with cross‐sectional analyzer plugin. The CSA value was evaluated only for muscle fibers (120 in number) contained entirely within the image. A blind independent operator performed histological analyses. All the morphological observations were adapted to those reported in previous studies on other fish models.^30^, ^31^

### Western blotting

WB experiments were carried out on the skeletal muscle of each zebrafish group (*n* = 6 zebrafish per group) by following protocols used in previous work.^26^ Total muscle proteins were extracted by using ice‐cold RIPA lysis buffer enriched with protease and phosphatase inhibitors. Proteins were quantified using a Bradford Protein Assay Kit (Thermo Scientific, USA), separated by 10% T SDS‐PAGE and membranes containing the electrotransferred proteins were immunoblotted with primary antibodies (Table S1). WB signals were quantified using Image Lab (Bio‐Rad) software.

### Proteomics

Skeletal muscle samples of zebrafish (*n* = 6 individuals per group) were sampled at the same location from two different fillets, pooled in equal amounts to obtain one replicate, and frozen in liquid nitrogen. Frozen muscle samples (40 mg) were homogenized with a polytron in 0.25–1 mL of 8.3 mol L^−1^ urea, 2 mol L^−1^ thiourea, 2% (w/v) CHAPS, and 1% (w/v) DTT, and resulting extracts were shaken for 30 min, at 4 °C, and centrifuged at 10 000 × *g* for 30 min. Proteins (100 μg) for each zebrafish group were reduced, alkylated with iodoacetamide, precipitated, and digested with trypsin as previously reported.^32^ Peptides obtained from each protein sample were labelled with the TMT Label Reagent Set (Thermo‐Fisher Scientific, USA) according to the matching CT‐TMT6‐126, I‐TMT6‐127, PV‐TMT6‐128, PVpreI‐TMT6‐129, PVwithI‐TMT6‐130, PVpostI‐TMT6‐131. Labeled peptide mixtures were prepared, pooled, and fractionated as already described.^32^ Eight fractions of TMT‐labelled peptides were collected and analyzed with an UltiMate 3000 HPLC RSLC nano system (Dionex, USA) linked to a Q‐ExactivePlus mass spectrometer, as previously reported.^32^ Technical triplicates were analyzed for all samples.

All mass spectrometry (MS) raw data files were merged in Proteome Discoverer software version 2.4 (Thermo Scientific) for identification and relative quantification of proteins, allowing database searching via Mascot algorithm v. 2.4.2 (Matrix Science, UK) of a UniProtKB protein database (*D. rerio*, 96 007 protein sequences, 12/2022) also including the most common protein contaminants. Additional database searching parameters were already reported.^32^ Protein candidates assigned based on at least two sequenced peptides and an individual Mascot Score greater than or equal to 25 were considered confidently identified. To calculate fold changes between samples, ratios of TMT reporter ion intensities in the MS/MS spectra from raw datasets were used. The results were filtered to obtain a false positive rate of 1%.

### Statistical analysis

Statistical analysis for histology and immunohistochemistry was performed using a one‐way analysis of variance (ANOVA) coupled with Bonferroni's *post hoc* test to identify differences between groups. Non‐normally distributed data, such as that obtained by WB, were evaluated by Kruskal–Wallis ANOVA nonparametric test followed by Dunn's *post hoc* test. GraphPad Prism 6 software v. 6.05 (GraphPad, Inc.) was used to perform statistical analyses; all the quantitative data were expressed as mean ± SD or mean ± SE. The significance level was set at *P <* 0.05.

## RESULTS

### Phytochemical quantitative profiling of chestnut shell and *Verbascum macrurum* extracts

The quantitative phytochemical characterization of the tested extracts revealed distinct, matrix‐specific bioactive profiles (Table 1). Chestnut (*C. sativa*) shell extract exhibited an exceptionally high content of total phenolics (412.50 ± 15.20 mg GAE g^−1^) and a substantial fraction of total hydrolyzable and condensed tannins (284.10 ± 11.40 mg g^−1^). Among the identified free phenolic constituents, hydroxybenzoic acids (65.30 ± 4.10 mg SAE g^−1^) and hydroxycinnamic acids (41.20 ± 2.80 mg FAE g^−1^) represented the primary fractions, with free gallic acid quantified at 21.90 ± 1.50 mg g^−1^, whereas flavonols were below the detection limit. Conversely, the *V. macrurum* extract showed a lower total phenolic content (185.40 ± 8.60 mg GAE g^−1^) compared to *C. sativa* counterpart (*P* < 0.05) but was uniquely characterized by a prominent, highly concentrated flavone fraction, with apigenin yielding the highest quantitative value (94.20 ± 5.30 mg LE g^−1^). Minor but significant amounts of ellagic acid (12.60 ± 0.90 mg g^−1^) and quercetin (8.40 ± 0.60 mg g^−1^) completed the *V. macrurum* bioactive profile, highlighting a complementary antioxidant and anti‐inflammatory biochemical composition between the two natural matrices selected for the dietary formulation.

**Table 1:** Phytochemical profile and quantitative analysis of chestnut (*C. sativa*) shell and *V. macrurum* extracts

Extract matrix	Phytochemical fraction/major bioactive compounds	Quantitative content (mg g^−1^ dry extract)	Detection wavelength/method
Chestnut shell	Total phenolics	412.50 ± 15.20ª	275 nm (GAE)
	Total tannins (hydrolyzable + condensed)	284.10 ± 11.40ª	Mass‐balance subtraction
	Hydroxybenzoic acids	65.30 ± 4.10ᵇ	260 nm (SAE)
	Hydroxycinnamic acids	41.20 ± 2.80ᶜ	325 nm (FAE)
	Free gallic acid	21.90 ± 1.50ᵈ	275 nm (external standard)
	Flavonols	ND	375 nm (kaempferol equivalents)
*Verbascum macrurum*	Total phenolics	185.40 ± 8.60ᵇ	280 nm (GAE)
	Apigenin	94.20 ± 5.30ª	280 nm (LE)
	Ellagic acid	12.60 ± 0.90ᶜ	280 nm (external standard)
	Quercetin	8.40 ± 0.60ᵈ	280 nm (external standard)

Results are expressed as mean ± SD (*n* = 3). Different superscript letters within the same extract matrix indicate statistically significant differences (*P* < 0.05). ND, not detected.

### Polyphenol effects on white muscle tissue morphology

Skeletal muscle from the CT group showed normal morphology, with compact fibers displaying apparent striations and peripheral oval nuclei (Fig. 1(A)). Inflammation caused notable alterations, including wavy, degenerated fibers lacking striations, broken myofibrils, some circular nuclei, and enlarged interstitial areas with occasional infiltrated cells (Fig. 1(B)). In the PVpreI group, most fibers appeared normal, though some degenerated ones without striations were seen (Fig. 1(C)). PVwithI muscle showed a morphology similar to that of the I group with wavy and degenerated fibers, missing striations, rare circular nuclei, broken myofibrils, and interstitial enlargements with infiltrating cells (Fig. 1(D)). PVpostI muscle displayed pronounced changes, including enlarged interstitial areas with cell infiltration, more connective tissue, wavy fibers – often without striations – and circular or central nuclei (Fig. 1(E)). The PV group showed muscle morphology comparable to that of control (Fig. 1(F)).

**Figure 1:**
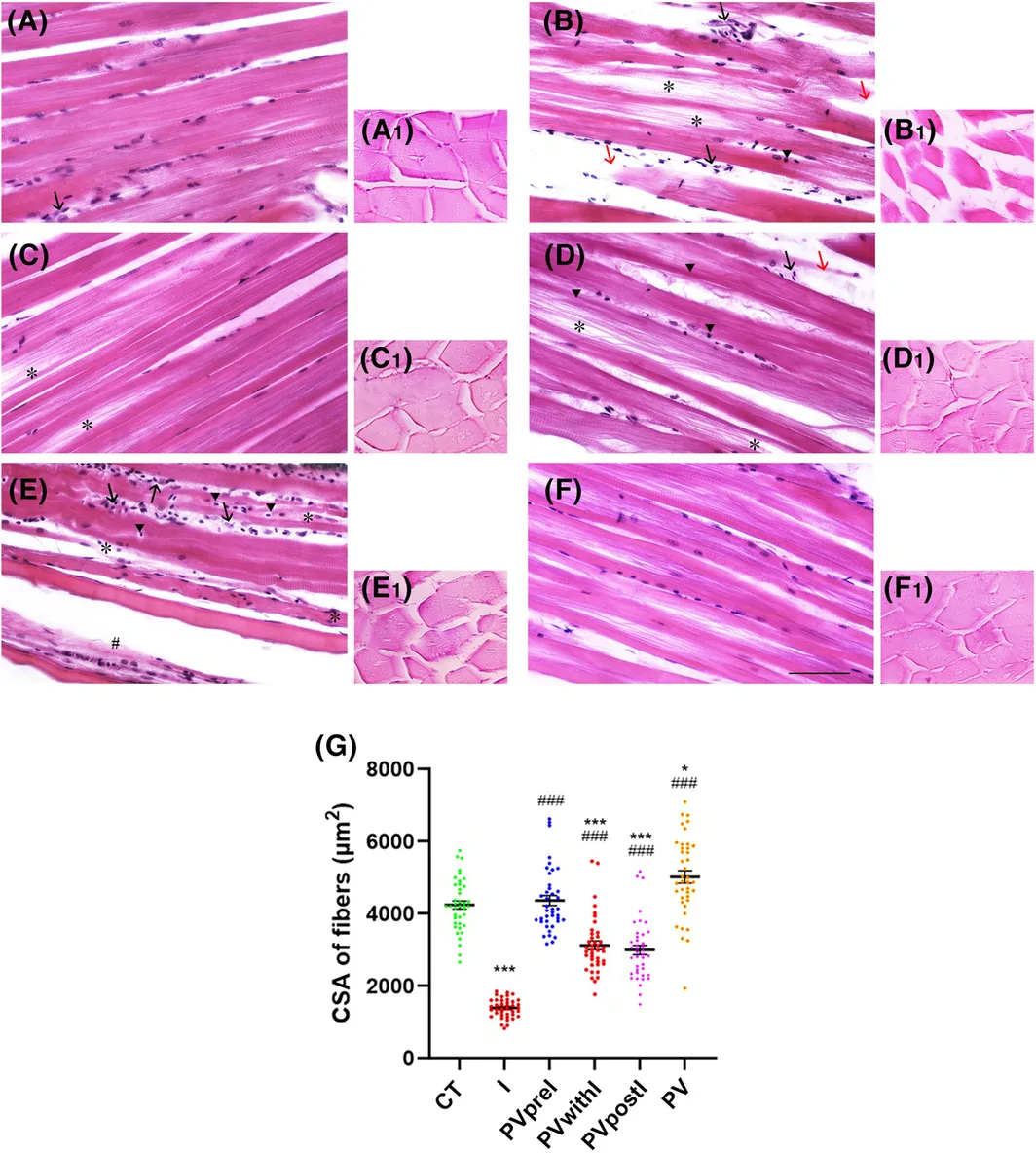
Representative images of white muscle tissue stained with hematoxylin–eosin. The images show muscle longitudinal and transversal sections of (A, A_1_) control (CT), (B, B_1_) inflamed (I), (C, C_1_) chestnut shell and *V. macrurum* polyphenols extract pre‐treated (PVpreI), or (D, D_1_) co‐treated (PVwithI), or (E, E_1_) post‐treated (PVpostI), and (F, F_1_) polyphenol‐treated control group (PV) zebrafish (*D. rerio*). Black arrowheads show circular nuclei; asterisks show degenerated and/or wavy fibers; black arrows show interstitial areas with cell infiltration; red arrows show broken myofibrillas; hash sign show increase in connective tissue spaces. Scale bar: 100 μm. (G) Mean muscle fiber CSA for each experimental group. Data are expressed as mean ± SE. ***P < 0.0001, *P < 0.05 compared to the CT group; ^###^ *P* < 0.0001 compared to the I group.

Analysis of transverse sections and CSA revealed enlarged inter‐fiber spaces in the I group and a significant decrease in mean CSA compared to CT (Fig. 1(G); *P* < 0.0001). PVpreI showed minimal interstitial space, and a significant CSA increase *versus* the I group (*P* < 0.0001), similar to CT. PVwithI had occasional interstitial enlargements and a CSA value significantly above that of the I group (*P* < 0.0001), but below that of CT (*P* < 0.0001) (Fig. 1(G)). PVpostI also showed enlarged interstitial spaces and a reduced CSA compared to CT (*P* < 0.0001), although still higher than that of the I group (*P* < 0.0001). The PV group showed a slight but significant CSA increase *versus* CT (*P* < 0.05).

### Polyphenol effects on TNF‐*α*, COX2, and caspase 3 immunoexpression in white muscle tissue

TNF‐*α* immunohistochemical staining of longitudinal skeletal muscle sections in the CT group showed absent or sporadic expression (Fig. 2(A)). In contrast, the I group displayed strong TNF‐*α* positivity in muscle fiber nuclei and infiltrated cells (Fig. 2(B)). In the PVpreI group, weak TNF‐*α* immunosignal appeared in a few muscle fiber nuclei (Fig. 2(C)), while the signal was more pronounced in PVpostI and PVwithI groups (Fig. 2(D),(E)). The PV group showed nearly absent TNF‐*α* expression, similar to CT (Fig. 2(F)).

**Figure 2:**
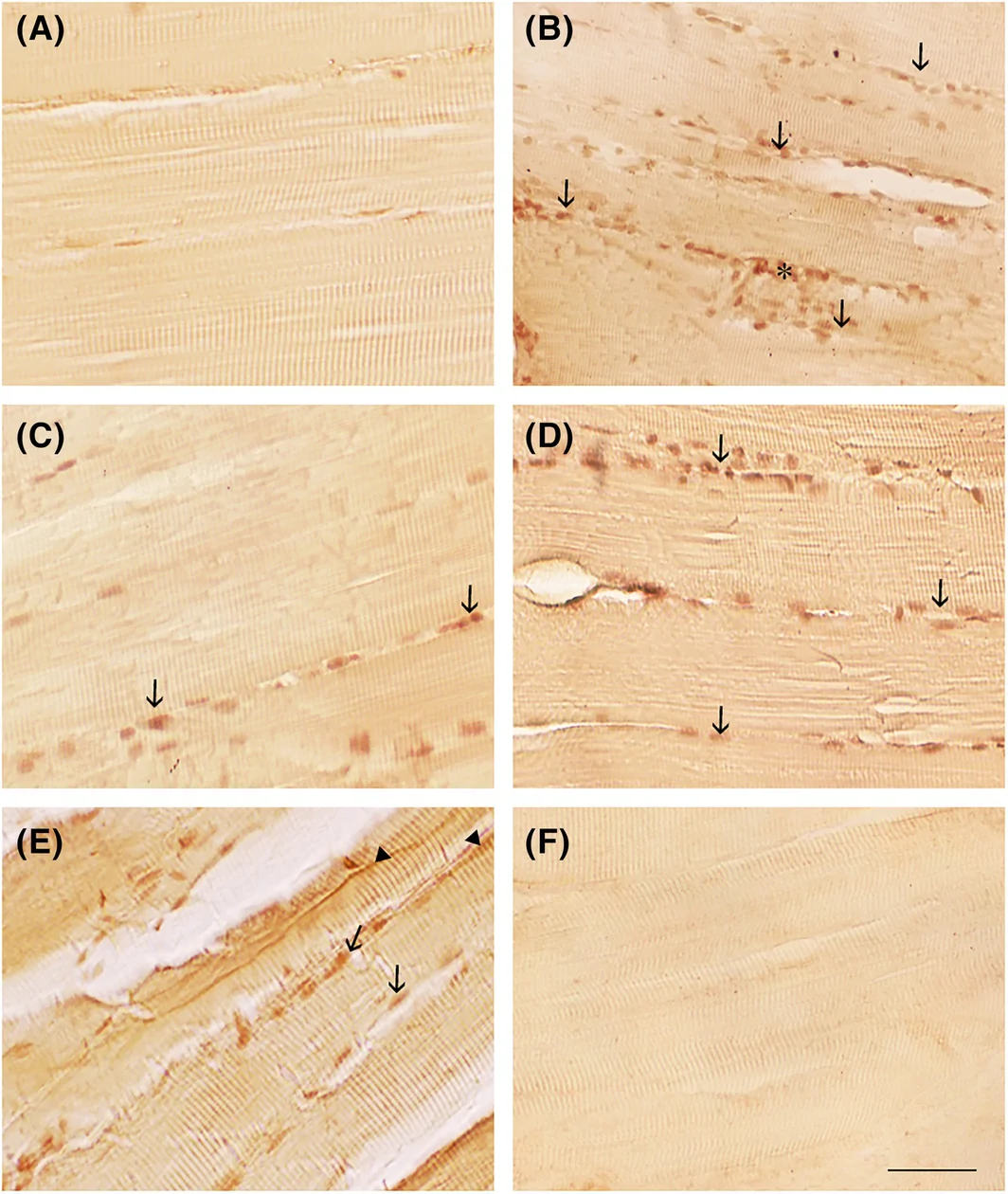
Representative images of TNF‐*α* immunoexpression in white muscle tissue. The images show TNF‐*α* immunosignal in muscle longitudinal sections of (A, A_1_) control (CT), (B, B_1_) inflamed (I), (C, C_1_) chestnut shell and *V. macrurum* polyphenols extract pre‐treated (PVpreI), or (D, D_1_) post‐treated (PVpostI), or (E, E_1_) co‐treated (PVwithI), and (F, F_1_) polyphenol‐treated control group (PV) zebrafish (*D. rerio*). Arrows show immunopositive nuclei of muscle cells; asterisks show immunopositive inflammatory infiltrated cells; arrowheads show immunopositive muscle fibers. Scale bar: 50 μm.

COX2 staining revealed a weak nuclear immunosignal in CT muscle fibers (Fig. 3(A)), while the I group showed strong COX2 positivity in both fibers and nuclei (Fig. 3(B)). No COX2 signal was detected in PVpreI muscle fibers (Fig. 3(C)), whereas some signals were observed in the nuclei of PVpostI (Fig. 3(D)) and muscle fibers of PVwithI (Fig. 3(E)). The PV group showed nearly absent COX2 expression (Fig. 3(F)).

**Figure 3:**
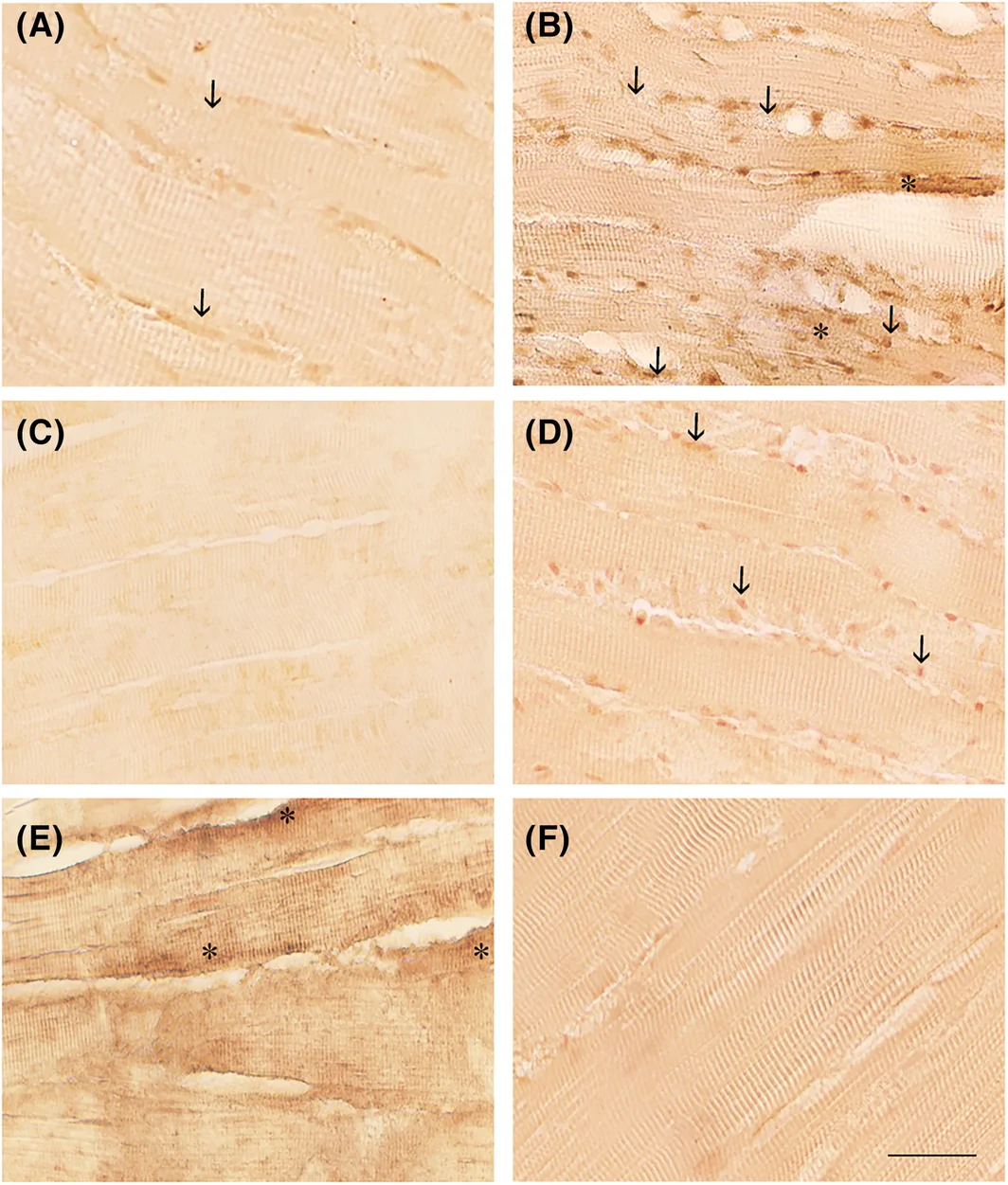
Representative images of COX2 immunoexpression in white muscle tissue. The images show COX2 immunosignal in muscle longitudinal sections of (A, A_1_) control (CT), (B, B_1_) inflamed (I), (C, C_1_) chestnut shell and *V. macrurum* polyphenols extract pre‐treated (PVpreI), or (D, D_1_) post‐treated (PVpostI), or (E, E_1_) co‐treated (PVwithI), and (F, F_1_) polyphenol‐treated control group (PV) zebrafish (*D. rerio*). Black arrows show immunopositive muscle cell nuclei; asterisks show immunopositive muscle fibers. Scale bar: 50 μm.

Caspase 3 immunostaining showed weak signal in CT muscle (Fig. 4(A)), but strong nuclear expression in I group fibers (Fig. 4(B)). PVpreI had limited nuclear positivity (Fig. 4(C)), while PVpostI and PVwithI showed increased nuclear caspase 3 signal (Fig. 4(D),(E)). The PV group had nearly no caspase 3 immunoexpression. Notably, the number of caspase 3‐positive nuclei was significantly higher in I (*P* < 0.05) and PVwithI (*P* < 0.0001) groups than in CT. PVpreI and PVpostI had significantly fewer positive nuclei than I group (*P* < 0.05), and levels comparable to that of CT.

**Figure 4:**
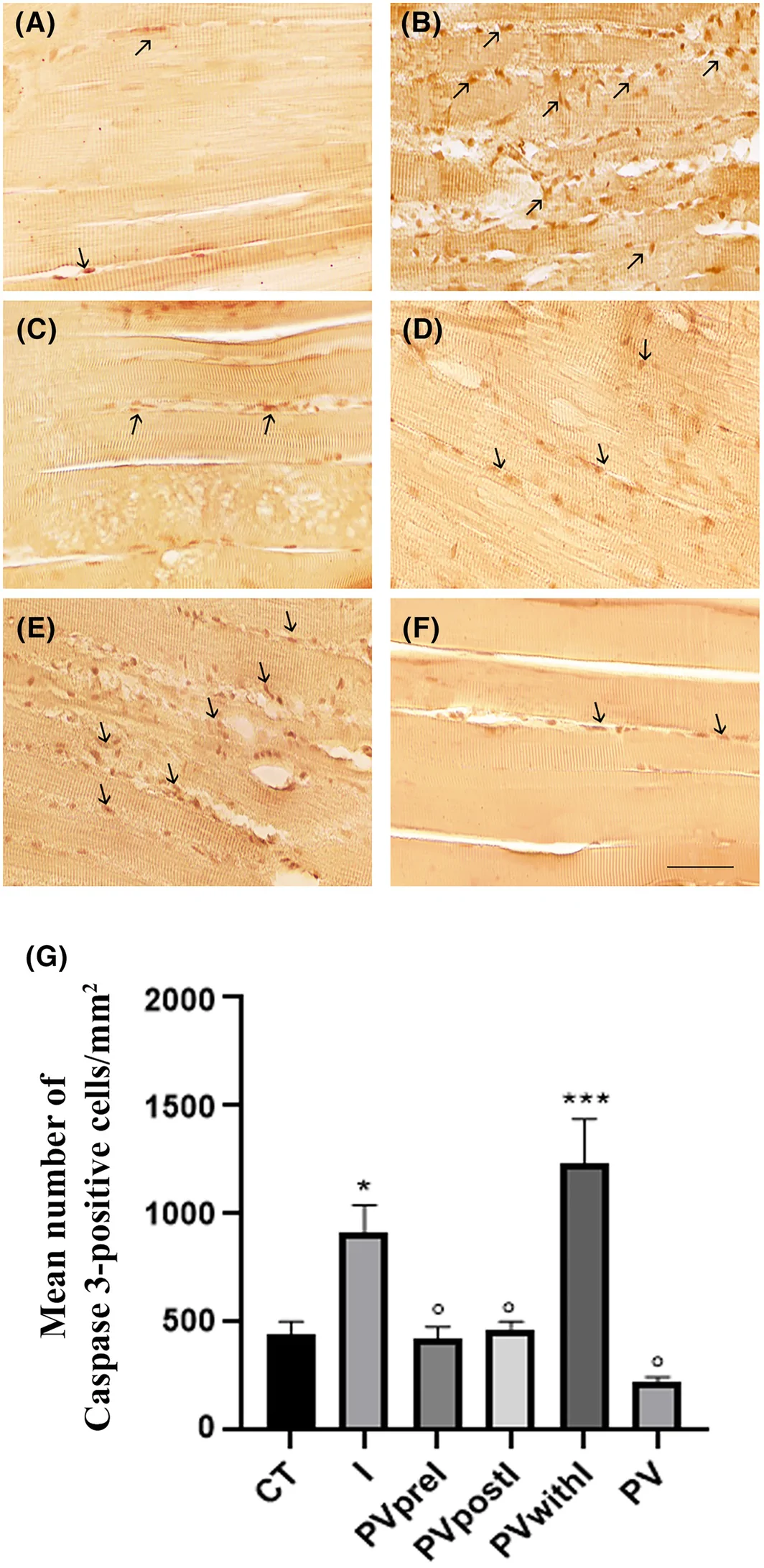
Representative images of caspase‐3 immunoexpression in white muscle tissue. The images show caspase‐3 immunosignal in muscle longitudinal sections of (A, A_1_) control (CT), (B, B_1_) inflamed (I), (C, C_1_) chestnut shell and *V. macrurum* polyphenols extract pre‐treated (PVpreI), or (D, D_1_) post‐treated (PVpostI), or (E, E_1_) co‐treated (PVwithI), and (F, F_1_) polyphenol‐treated control group (PV) zebrafish (*D. rerio*). Arrows show immunopositive nuclei of muscle cells. Scale bar: 50 μm. (G) Mean number of caspase‐3‐positive cells mm^−2^ of muscle tissue of each group. Data are expressed as mean ± SE. ***P < 0.0001, *P < 0.05 compared to the CT group; °*P* < 0.05 compared to the I group.

### Polyphenol effects on NF‐*κ*B and p38/MAPK, ERK 1/2 signaling pathway in white muscle tissue

I*κ*B*α* phosphorylation was assessed in skeletal muscle extracts from all experimental groups (Fig. 5). Inflammation (*P <* 0.05) and the PVpostI condition (*P <* 0.001) significantly increased the pI*κ*B*α*/I*κ*B*α* ratio compared to CT. In contrast, PVpreI (*P <* 0.001) and PVwithI (*P <* 0.05) significantly reduced pI*κ*B*α* levels *versus* PVpostI, with no differences from CT.

**Figure 5:**
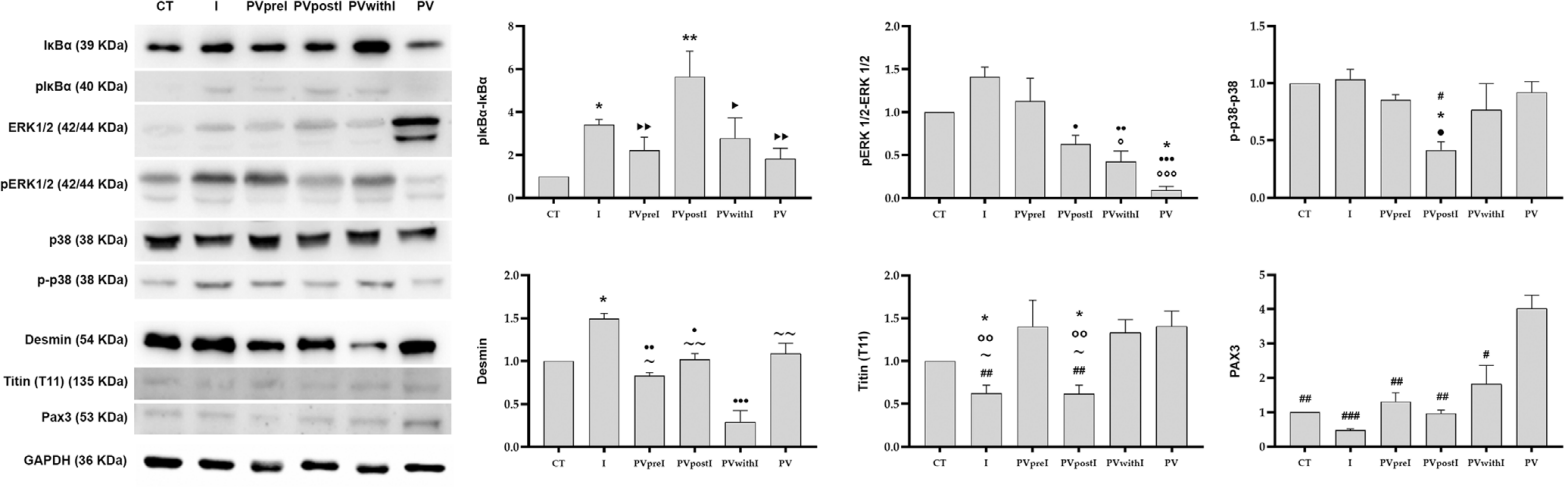
Representative immunoblots of I*κ*B*α* (39 kDa), phospho‐I*κ*B*α* (40 kDa), ERK ½ (42/44 kDa), phospho‐ERK ½ (42/44 kDa), p38 (38 kDa), phospho‐p38 (38 kDa), desmin (54 kDa), titin (T11) (135 kDa), and PAX3 (53 kDa) expression. Fold data of relative optical density are expressed as the ratios phospho‐I*κ*B*α*–I*κ*B*α*, phospho‐ERK 1/2–ERK 1/2, phospho‐p38–p38, and optical density of desmin, titin (T11), and PAX3 total protein signals in white muscle tissue of control (CT), inflamed (I), chestnut shell and *V. macrurum* polyphenols extract pre‐treated (PVpreI), or post‐treated (PVpostI), or co‐treated (PVwithI), and polyphenol‐treated control group (PV) zebrafish (*D. rerio*). The relative amount of each protein was compared with that of GAPDH (36 kDa). Fold data are presented as the mean ± SD from *n* = 3 zebrafish per group, normalized to the CT group. **P < 0.001, *P < 0.05 compared to the CT group; •••*P* < 0.0001, ••*P* < 0.001, •*P* < 0.05 compared to the I group; 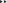 *P* < 0.001,  *P* < 0.05 compared to the PVpostI group; °°°*P* < 0.0001, °°*P* < 0.001, °°*P* < 0.05 compared to the PVpreI group; ^~~^ *P* < 0.001, ^~^ *P* < 0.05 compared to the PVwithI group; ^###^ *P* < 0.0001, ^##^ *P* < 0.001, ^#^ *P* < 0.05 compared to the PV group.

For MAPK signaling, PVwithI (*P <* 0.001) and PVpostI (*P <* 0.05) showed significantly reduced ERK 1/2 phosphorylation compared to the I group. The PV group exhibited the lowest ERK 1/2 phosphorylation. No increase in p38/MAPK phosphorylation was observed in I *versus* CT (Fig. 5), and only PVpostI showed significantly lower p38/MAPK levels compared to I (*P <* 0.05).

### Polyphenol effects on desmin, titin, and PAX3 in white muscle tissue

As shown in Fig. 5, desmin levels were significantly higher in the I group compared to CT (*P* < 0.05). In PVpreI, PVpostI, and PVwithI groups, desmin levels were similar to that of CT, even if PVwithI showed the lowest amount. Titin (segment T11) levels were significantly reduced in I and PVpostI compared to CT (*P* < 0.05), while PVpreI, PVwithI, and PV showed levels comparable to that of CT (Fig. 5). PAX3 expression remained unchanged in I and all polyphenol‐treated groups (PVpreI, PVpostI, PVwithI) but significantly increased in the PV group (Fig. 5).

### Polyphenol effects on white muscle proteomic repertoire

Tissue protein extracts from CT, I, PVpreI, PVpostI, PVwithI, and PV groups were independently pooled and analyzed via TMT‐label quantitative proteomics combined with nLC‐ESI‐Q‐Orbitrap‐MS/MS, defining the proteomic signature of each diet (Fig. S1). This high‐resolution approach identified and quantified 2477 unique proteins in zebrafish white muscle. Proteomic data were filtered for a fold change of 0.66–1.5 and *P* < 0.05 in pairwise comparisons, identifying differentially represented proteins (DRPs). A total of 251 DRPs were found in the following comparisons: (i) I *versus* CT (39 over‐ and 15 down‐represented; Fig. S2(A)); (ii) PV *versus* CT (33 over‐, 47 down‐represented; Fig. S2(B)); (iii) PVpostI *versus* I (40 over‐, 72 down‐represented; Fig. S2(C)); (iv) PVpreI *versus* I (30 over‐, 84 down‐represented; Fig. S2(D)); (v) PVwithI *versus* I (24 over‐, 127 down‐represented; Fig. S2(E)). Table S2 lists all DRPs with related annotations, peptides, post‐translational modifications, and quantitation data.

To explore DRP relationships, a protein–protein interaction analysis via STRING^33^ revealed an extensive interconnected network of 208 DRPs, alongside smaller subnetworks of 2–5 proteins (Fig. S3), suggesting strong functional links. Gene Ontology enrichment analysis (Fig. 6) using Metascape and EnrichR indicated DRP involvement in muscle functions (e.g. structure development), inflammation, immune response, oxygen/metabolite transport, and metabolic processes.

**Figure 6:**
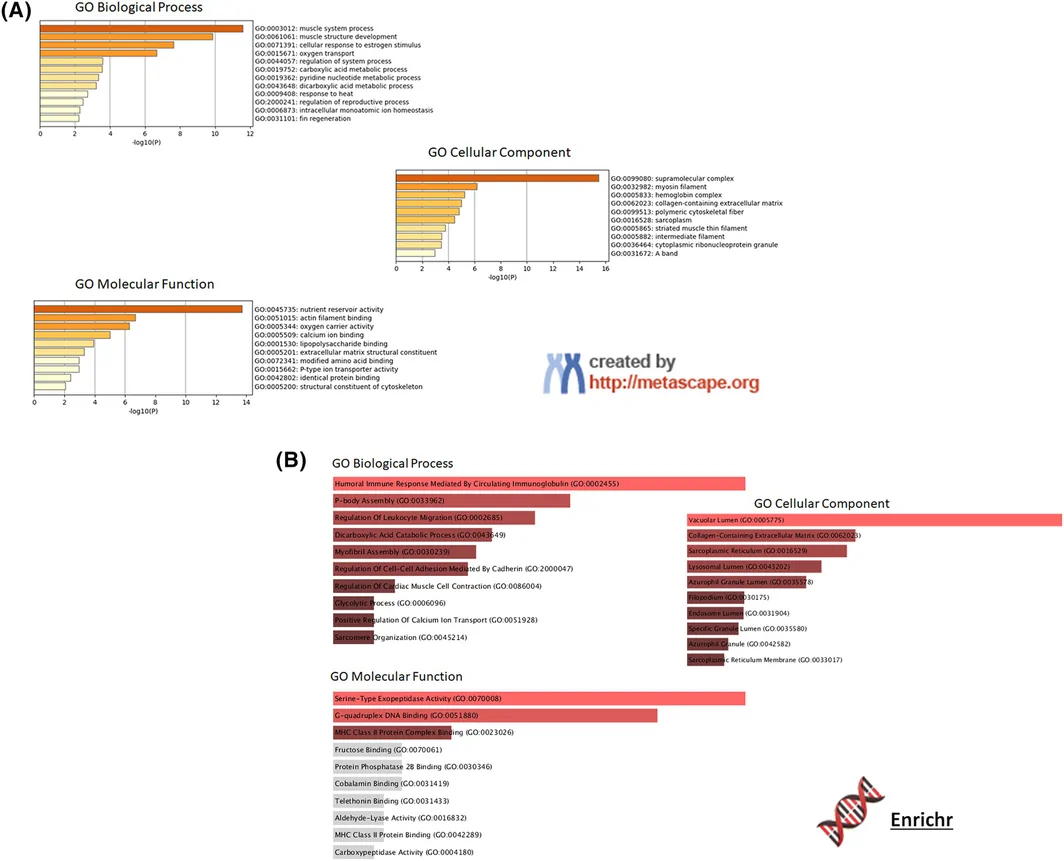
Protein enrichment analysis of DRPs observed in skeletal muscle of zebrafish experiencing different diet regimens as deduced by the Metascape tool (A) and by the EnrichR tool (B). (A) The analysis identifies enriched biological pathways, molecular functions, and cellular components associated with observed DRPs. The enrichment terms are visualized as a bar graph, with the size of each bar representing the number of DRPs annotated to each term and the color reflecting the statistical significance of the enrichment. (B) The analysis identifies enriched key biological processes, molecular functions, and cellular components associated with observed DRPs. The enrichment terms are visualized as a bar graph, with the size of each bar representing the number of DRPs annotated to each term and the color reflecting the statistical significance of the enrichment.

Further filtering excluded DRPs unique to single comparisons, resulting in 127 proteins showing changes across multiple comparisons: 41 in two, 48 in three, 27 in four, and 11 in all five (Figs 7 and 8). These included structural proteins and regulators (20%), inflammation and host defense proteins (19%), metabolite transporters (13%), connective tissue components (9%), morphogenesis/development‐related proteins (6%), proteases and inhibitors (5%), energy metabolism components (5%), glycolytic enzymes (3%), signaling proteins (3%), DNA/RNA processing proteins (3%), and those involved in amino acid (3%) or lipid metabolism (2%), chaperones (2%), unknown function (5%), and others (2%). Many proteins showed consistent changes in PVpreI, PVwithI, and PVpostI *versus* I, with opposite trends in I *versus* CT (Figs 7 and 8).

**Figure 7:**
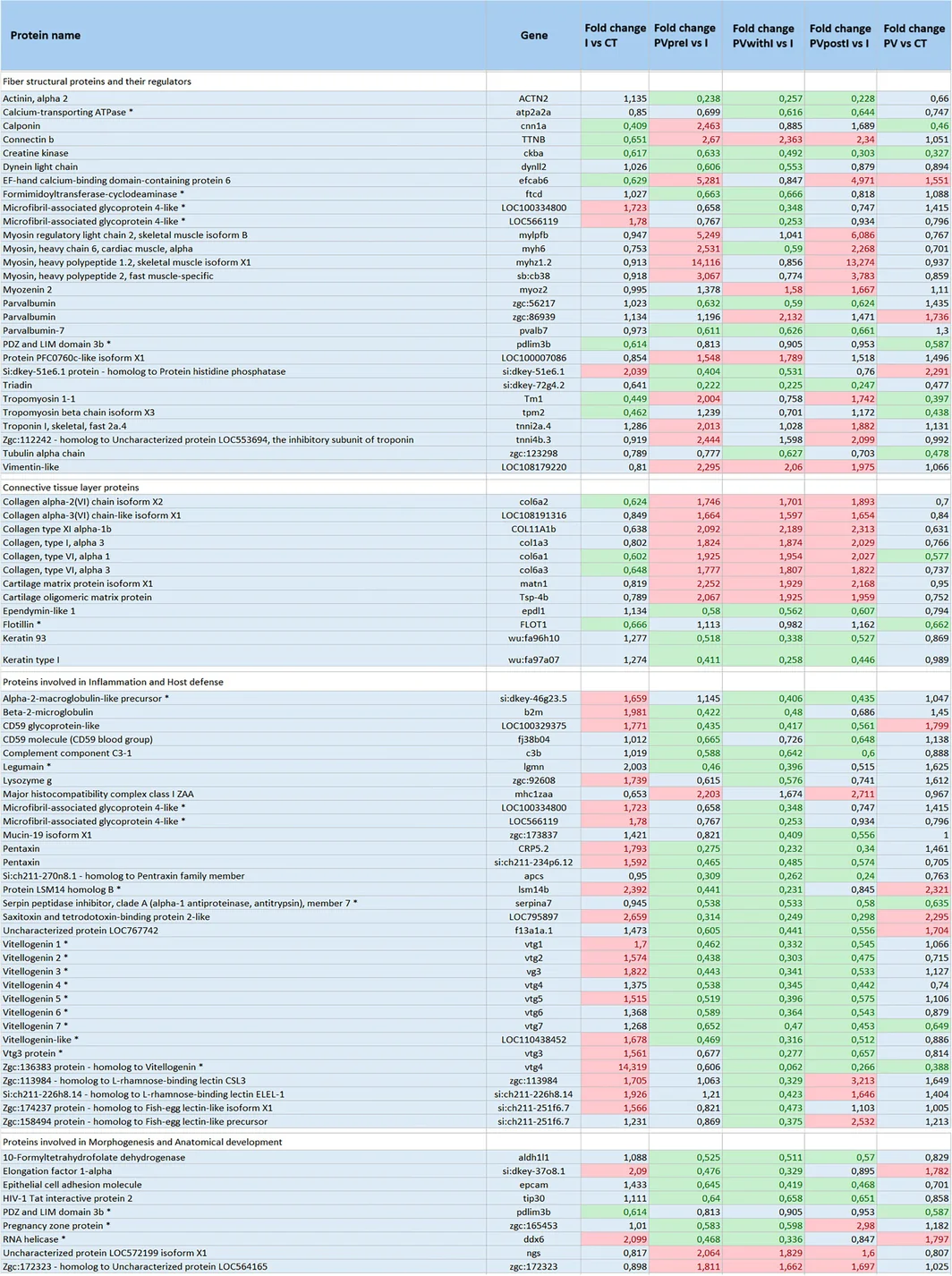
DRPs assigned in at least two of the I *versus* CT, PVpreI *versus* I, PVwithI *versus* I, PVpostI *versus* I and PV *versus* CT comparisons. Proteins are grouped according to their function. Fiber structural proteins and their regulators; connective tissue layer proteins; proteins involved in inflammation and host defense; proteins involved in morphogenesis and anatomical development are reported. Proteins showing multiple functions are indicated with an asterisk; for simplicity, no more than two functions are considered. Highlighted in color are the fold change values for which a concomitant 0.66 ≥ fold change ≥ 1.5 and an abundance ratio *P* <0.05 in each pairwise comparison were observed. Although not concomitantly satisfying both above‐reported criteria, additional proteins within the PVpreI *versus* I, PVwith *versus* I, and PVpostI *versus* I comparisons showed an opposite quantitative trend with respect to that observed in the I *versus* CT comparison, thus confirming the general positive effect of pre‐, co‐ and post‐treatments with dietary polyphenols of animals experiencing the pro‐inflammatory diet in recovering the proteomic variations induced by the latter diet with respect to control. These proteins were not highlighted in red and green color but showed coherent fold change values.

**Figure 8:**
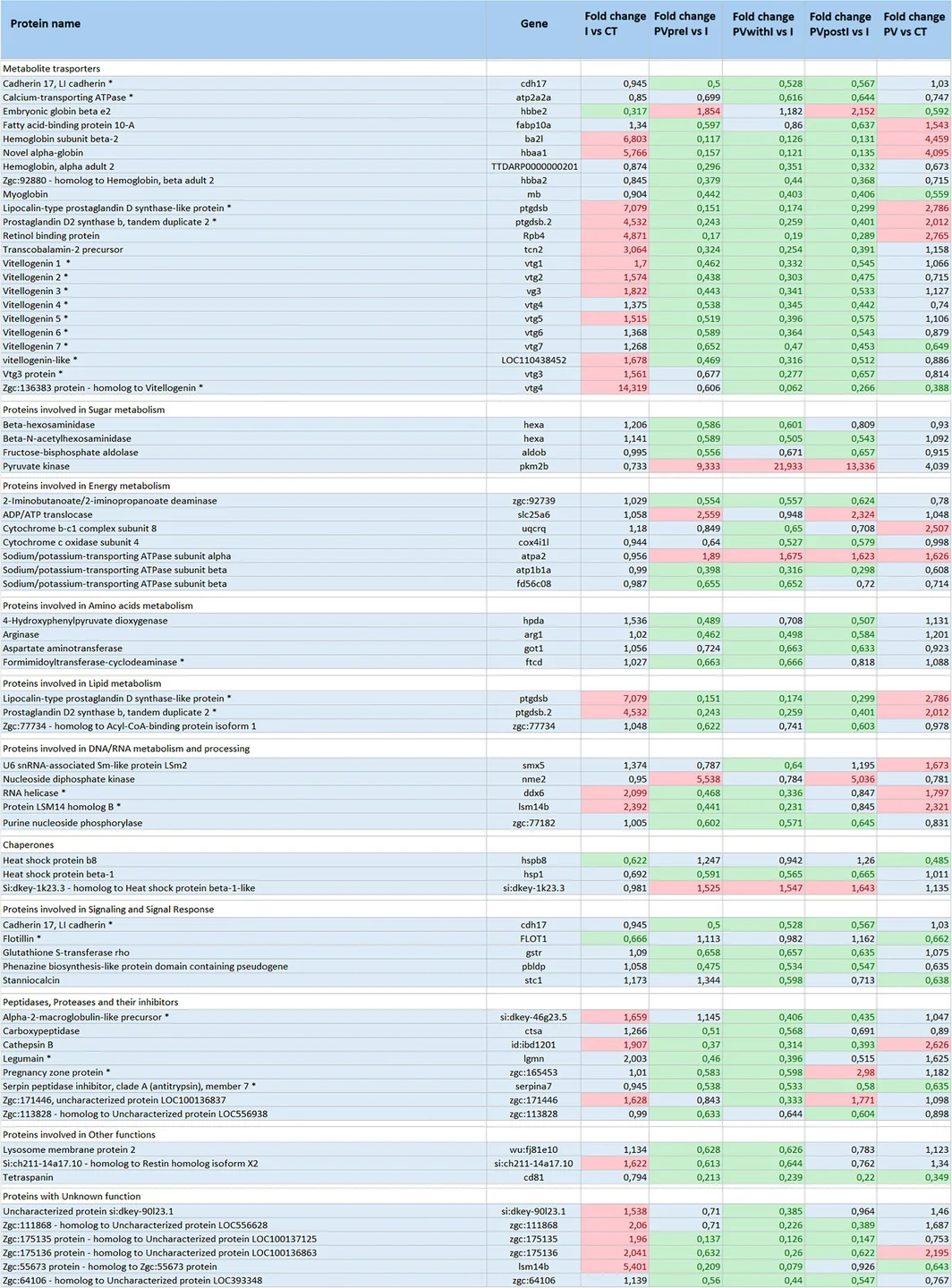
DRPs assigned in at least two of the I *versus* CT, PVpreI *versus* I, PVwithI *versus* I, PVpostI *versus* I, and PV *versus* CT comparisons. Proteins are grouped according to their function. Metabolite transporters, proteins involved in sugar, energy, amino acids and lipid metabolism; proteins involved DNA/RNA metabolism and processing; chaperones; proteins involved in signaling and signal response; peptidases, proteases, and their inhibitors; proteins involved in other functions; proteins with unknown functions are reported. Proteins showing multiple functions are indicated with an asterisk; for simplicity, no more than two functions were considered. Highlighted in color are the fold change values for which a concomitant 0.66 ≥ fold change ≥ 1.5 and an abundance ratio *P* < 0.05 in each pairwise comparison were observed. Although not concomitantly satisfying both above‐reported criteria, additional proteins within the PVpreI *versus* I, PVwith *versus* I, and PVpostI *versus* I comparisons showed an opposite quantitative trend with respect to that observed in the I *versus*. CT comparison, thus confirming the general positive effect of pre‐, co‐ and post‐treatments with dietary polyphenols of animals experiencing the pro‐inflammatory diet in recovering the proteomic variations induced by the latter diet with respect to control. These proteins were not highlighted in red and green color but showed coherent fold change values.

## DISCUSSION

This study reports the reversing effects of polyphenol blend administered before, during, and after a pro‐inflammatory diet on zebrafish skeletal muscle morphology and protein expression. The polyphenol mix used included condensed tannins (epigallocatechin and catechin/epicatechin) from chestnut shell^28^, ^34^ and flavonoids (mainly apigenin) from *V. macrurum*.^26^

In inflamed zebrafish, muscle morphology was notably altered. Fibers appeared disassembled or broken, with absent striations and enlarged interstitial areas with cell infiltration, hallmarks of atrophic muscle.^35^, ^36^ These changes were linked to alterations in structural muscle proteins. Proteomic data revealed reduced levels of calponin, connectin b, EF‐hand Ca^2+^‐binding protein 6, PDZ and LIM domain 3b, and specific tropomyosin isoforms, while microfibril‐associated glycoprotein 4‐like isoforms and histidine phosphatase were increased (I *versus* CT). Polyphenol pre‐treatment prevented most morphological and proteomic changes, while co‐ and post‐treatments were partially effective.

Focusing on titin, a key contractile protein spanning the sarcomere, WB analysis showed reduced titin segment T11 in inflamed muscle, indicating partial degradation. Pre‐ and co‐treatments mitigated this reduction, unlike post‐treatment. Proteomic data also showed that connectin b, a titin a‐derived fragment involved in elasticity,^6^, ^37^, ^38^ decreased in the I group and increased following all polyphenol treatments. These findings suggest that polyphenols influence titin turnover, although more research is needed to clarify their exact role.

Tropomyosin isoforms decreased in the I group and were restored mainly by pre‐treatment. Tropomyosin stabilizes actin filaments and regulates their dynamics.^39^ Interestingly, green tea polyphenols have been shown to modify tropomyosin conformation in shrimp,^40^ but such a possibility remains unexplored in vertebrates.

Though not differing between I and CT groups, polyphenol treatments affected the abundance of other muscle proteins: actinin 2, Ca^2+^‐transporting ATPase, dynein light chain, formimidoyltransferase‐cyclodeaminase, tubulin, triadin, parvalbumin isoforms, myozenin 2, vimentin‐like protein, troponins, and various myosin isoforms. These proteins regulate actomyosin function, contraction, and cytoskeletal integrity^41^ and have been shown to respond to flavonoids such as apigenin, epigallocatechin, and quercetin.^42^

WB analysis also revealed desmin overexpression in inflamed muscle, which was reversed by all polyphenol treatments. Desmin connects myofibrils and anchors them to membranes and organelles.^15^ Its overexpression is linked to ageing and inflammation, resulting in pathological protein aggregates.^15^, ^43^

The reduction in CSA and appearance of large inter‐fiber spaces in inflamed muscle reflected muscle atrophy.^44^ In zebrafish and other animals, these spaces are typically filled by collagen‐rich extracellular matrix.^44^ Polyphenol treatments prevented and partially reversed CSA reduction. Consistently, flavonoid‐rich soy improved CSA in obese rats.^45^

The muscle is covered by layers of connective tissue called epimysium, perimysium, and endomysium.^46^ Inflammation affects collagen expression variably across models.^47^, ^48^ Proteomics showed that polyphenol treatments led to over‐representation of multiple collagen isoforms, aligning with green tea polyphenols enhancing collagen synthesis in murine cardiac tissue.^49^ Cartilage oligomeric matrix protein and cartilage matrix protein isoform X1 were increased in polyphenol‐treated inflamed zebrafish, while ependymin‐like 1 and some keratins decreased. Cartilage oligomeric matrix protein has been linked to polyphenol‐rich diets in murine arthritis models.^50^

Inflammation, key to muscle regeneration, involves TNF‐*α*, produced by immune and muscle cells, which regulates regeneration via p38 activation.^51^ TNF‐*α* expression rose in inflamed zebrafish but decreased after pre‐treatment, although p38 activation was not observed. Similarly, COX2, an enzyme driving prostaglandin‐mediated inflammation,^4^ was elevated in inflamed muscle and reduced by polyphenol treatment. Curcumin and other polyphenols inhibit NF‐*κ*B signaling and decrease pro‐inflammatory cytokines.^52^, ^53^, ^54^ WB data showed reduced I*κ*B*α* phosphorylation in pre‐ and co‐treated groups, aligning with studies on caffeic acid and resveratrol.^53^, ^54^

TNF‐*α* and COX2 expression changes paralleled proteomic shifts in immune‐related proteins in I *versus* CT: *α*‐2‐macroglobulin‐like precursor, *β*‐2‐microglobulin, CD59, lysozyme g, microfibril‐associated glycoproteins, pentaxins, LSM14, saxitoxin/tetrodotoxin‐binding proteins, rhamnose‐binding lectins, and vitellogenins – all reverted by polyphenol treatment. These proteins modulate inflammation, immunity, and phagocytosis, confirming polyphenols' immunomodulatory potential. Rhamnose‐binding lectins, found in various teleosts including zebrafish, are key regulators of innate immunity.^55^, ^56^, ^57^

ERK, crucial for muscle stem cell proliferation,^58^ was upregulated in inflamed muscle but reduced after pre‐ and post‐treatment. Quercetin was previously shown to reduce ERK, p38, and NF‐*κ*B activation in obese mice with muscle atrophy.^59^ Related to regeneration, PDZ‐LIM domain 3b was reduced in the I group, while elongation factor 1*α* and DDX6 increased; these effects were reversed by pre‐ and co‐treatment. Both proteins are involved in proliferation and development, with elongation factor 1*α* playing a key role in zebrafish tissue regeneration.^60^

Interestingly, diet‐induced inflammation triggered a robust activation of the NF‐*κ*B and ERK 1/2 pathways, in the inflamed group compared to control, while no significant changes were observed for p38/MAPK phosphorylation in the inflamed group compared to the control. This discrepancy may be attributed to the distinct activation kinetics of these signaling cascades; p38/MAPK is typically involved in early, acute stress responses in skeletal muscle. In a 10‐day sub‐chronic dietary model, its transient activation might have already returned to baseline, whereas NF‐*κ*B and ERK 1/2 remain sustained to drive progressive muscle remodeling.^61^, ^62^ Notably, the significant downregulation of p38 phosphorylation in the PVpostI group suggests a targeted ability of the polyphenol blend to suppress this regulatory axis during the recovery phase, maybe by acting on pro‐atrophic transcription factors, which are known downstream targets of prolonged p38 signaling in wasting muscle.^63^

Metabolite transporters shape immune cell activity.^61^, ^62^ Inflamed zebrafish showed over‐representation of hemoglobin subunits, prostaglandin D synthase‐like isoforms, retinol‐binding protein, transcobalamin‐2, and vitellogenins, reversed by polyphenol treatment. Changes in retinol‐binding protein agree with findings from green tea polyphenol‐fed insulin‐resistant rats.^63^

No significant enzyme deregulation in sugar, energy, or amino acid metabolism was seen in I *versus* CT, suggesting minimal metabolic disruption. However, polyphenol treatments did modulate proteins such as cytochrome b‐c1 complex subunit 8, cytochrome c oxidase subunit 4, and ATPase subunits – supporting a role in mitochondrial regulation.^64^ Polyphenol‐treated groups also showed lower aspartate aminotransferase levels, consistent with previous rat studies.^65^ Only a few lipid and nucleic acid metabolism‐related proteins, including prostaglandin D2 synthase b, DDX6, and LSM14, changed with inflammation but were normalized by polyphenol treatments. Others – acyl‐CoA‐binding protein, nucleoside diphosphate kinase, and purine nucleoside phosphorylase – responded independently to polyphenol treatments. Effects on metabolic pathways were less pronounced than those on structural or immune proteins, likely due to polyphenols' modulatory roles.^66^

Although the canonical muscle‐protective mechanisms of polyphenols have been extensively mapped in standard atrophy or high‐fat diet models,^15^ our study establishes, for the first time, a direct *in vivo* link between diet‐induced intestinal inflammation and downstream skeletal muscle degeneration, highlighting the efficacy of dietary polyphenol blend via the functional gut–muscle axis. Our untargeted proteomic workflow expands beyond standard biomarkers, revealing that polyphenols protect myofibrillar architecture through isoform‐specific regulation of structural targets, including titin (T11 segment), connectin b, tropomyosin, and desmin. Finally, we demonstrate that polyphenol efficacy is strictly time‐dependent, transitioning from a superior protective effect induced by pre‐treatment to a less active tissue recovery (post‐treatment). Specifically, exposing zebrafish to the polyphenol blend prior to k‐carrageenan‐induced inflammation may cause the accumulation of bioactive molecules in tissues, a process capable of stimulating the nuclear translocation of nuclear factor erythroid 2‐related factor 2, a key regulator of cellular antioxidant defense and inflammation.^64^, ^67^ Consequently, when the inflammatory stimulus is subsequently introduced, the tissue is already primed to suppress the activation of key inflammatory centers such as NF‐*κ*B, preserving structural integrity at a preventive level. In contrast, co‐treatment offers immediate mitigation against acute oxidative and inflammatory waves, while post‐treatment interfaces with an already damaged tissues; at this late stage, polyphenols promote structural resolution rather than prevention, uniquely downregulating p38/MAPK phosphorylation to shut down residual stress pathways and accelerate muscle remodeling. These temporal layers significantly advance the molecular understanding of how complex natural extracts systemically counteract diet‐dependent muscle atrophy.

In summary, our results suggest that the previously observed reduction of intestinal inflammation induced by a polyphenol‐enriched diet^26^ likely counteracts the systemic inflammatory cascade and prevents skeletal muscle atrophy by favorably modulating the muscle proteome and restoring protein composition. However, it is worth noting that, although our primary goal was to evaluate a preventive dietary nutritional strategy rather than a pharmacological treatment, the comparison of natural polyphenols with known anti‐inflammatory or anti‐atrophic compounds will represent an important step in future studies to accurately assess the relative efficacy of this bioactive blend.

## CONCLUSION

This study shows that a pro‐inflammatory diet induces profound structural, inflammatory, and proteomic alterations in zebrafish skeletal muscle, while a dietary polyphenol blend (tannins + apigenin) prevented or mitigated these detrimental effects. By modulating the balance of protein synthesis/degradation, immune response, and regeneration pathways, these bioactive compounds preserve muscle fiber integrity and provide a mechanistic basis for nutritional interventions. Overall, these findings support dietary polyphenol supplementation as a potential strategy to bridge the gap between high‐quality aquaculture production and the development of nutraceutical‐based therapies to combat sarcopenia and muscle wasting associated with chronic inflammatory diseases. However, proper administration timing is critical to prevent or ameliorate diet‐induced muscle damage.

## AUTHOR CONTRIBUTIONS

Conceptualization: Marina Paolucci and Roberta Imperatore; Data curation: Graziella Orso, Monica Matuozzo, Chiara D'Ambrosio, and Roberta Imperatore; Formal analysis: Graziella Orso and Chiara D'Ambrosio; Investigation: Graziella Orso, Monica Matuozzo, Seyed Hossein Hoseinifar, Ghasem Ashouri, Chiara D'Ambrosio, and Roberta Imperatore; Methodology: Graziella Orso, Mariapina Rocco, Ghasem Ashouri, Chiara D'Ambrosio, and Roberta Imperatore; Supervision: Andrea Scaloni, Marina Paolucci, and Roberta Imperatore; Validation: Marina Paolucci and Roberta Imperatore; Writing – original draft: Andrea Scaloni, Marina Paolucci, Chiara D'Ambrosio, and Roberta Imperatore; Writing – review & editing: Andrea Scaloni, Marina Paolucci, and Roberta Imperatore. All authors have read and agreed to the published version of the manuscript.

## FUNDING INFORMATION

This research received no external funding. This study was supported by internal institutional research funds from the University of Sannio, Fondi Ricerca di Ateneo (FRA).

## ETHICS STATEMENT

The authors confirm that the ethical considerations were met. This study has been done under Iran National Research Council's guidelines for the care and use of laboratory animals. The research board of GUASNR approved the main project on zebrafish as well as ethical considerations, no. 96‐374‐63.

## CONFLICT OF INTEREST

The authors declare no conflicts of interest. A previous version of the paper is online as a preprint (https://www.preprints.org/manuscript/202508.1585).

## Supporting information


**Figure S1.** Scheme of the TMT‐label based proteomic approach used for quantitative analysis of differentially represented proteins (DRPs) in the skeletal muscle tissues of zebrafish experiencing different diet regimens. The scheme shows the different diet regimens (CT, control animals; I, animals experiencing the pro‐inflammatory diet; PVpreI, animals experiencing pre‐treatment with chestnut shell polyphenols and *V. macrurum* extract before the proinflammatory diet; PVwithI, animals experiencing co‐treatment with chestnut shell polyphenols and *V. macrurum* extract and the proinflammatory diet; PVpostI, animals experiencing the proinflammatory diet followed by post‐treatment with chestnut shell polyphenols and *V. macrurum* extract; PV, control animals fed with chestnut shell polyphenols and *V. macrurum* extract). The proteomic workflow includes sample preparation, TMT labeling, mass spectrometry analysis by nLC‐ESI‐Q‐Orbitrap‐MS/MS followed by protein identification and quantification by dedicated bioinformatics.
**Figure S2.** Volcano Plot of DRPs observed in the skeletal muscle of zebrafish experiencing different diet regimens as revealed by analysis with Proteome Discoverer software of mass spectrometric data. The Volcano plot shows the distribution of differentially represented proteins in each pairwise comparison. Significantly over‐ and down‐represented proteins (Log_2_FC > 0.58, *P* < 0.05) are reported in highlighted red and green areas, respectively, while components with non‐significant differences are shown in the grey region. Abbreviations related to the different diet regimens are described in Figure S1.
**Figure S3.** STRING analysis of DRPs observed in the skeletal muscle of zebrafish experiencing different diet regimens. STRING analysis was performed with medium confidence to highlight protein interaction network of the differentially represented components ascertained in this study, providing insights into their potential biological roles and functional associations.

**Table S1.** Western Blotting primary antibodies.

**Table S2.** Differentially represented proteins (DRPs) identified in I *versus* CT, PVpreI *versus* I, PVwithI *versus* I, PVpostI *versus* I and PV *versus* CT comparisons.

## Data Availability

The datasets produced and analyzed during the current study are included in the article/Supplementary Materials. Further inquiries can be directed at the corresponding author.
