# Supplementary material for: Polyphenol dietary supplementation prevents inflammation‐induced muscle atrophy in a zebrafish ( Danio rerio ) model
Source: J Sci Food Agric. 2026 Jul 25;106(15):9581–95. doi: 10.1002/jsfa.70932 (PMC13743393; doi:10.1002/jsfa.70932)
Supplement: Supplementary file 1 [file JSFA-106-9581-s001.docx]

**Supplemental Tables**

**Table S1: Western Blotting primary antibodies**

| Primary Antibodies | Catalog Number | Company |
| --- | --- | --- |
| p44/42 MAPK (ERK 1/2) | #9107 | Cell Signaling |
| Phospho-p44/42 MAPK (ERK 1/2) (Thr202/Tyr204) | #9101 | Cell Signaling |
| IκBα | #55481S | ANASPEC |
| Phospho-IκBα (Ser32/36) | #9246 | Cell Signaling |
| p38 | #orb315599 | Biorbyt |
| Phospho-p38 MAPK (Thr180/Tyr182) | #NB500-138 | Novusbio |
| PAX3 | #MBS3201130 | MyBioSource |
| Desmin | #orb379480 | Biorbyt |
| Titin (connectin) | #NB600-1206 | Novus Biologicals |
| GAPDH | #Ab210113 | Abcam |

**Table S2.** **Differentially represented proteins (DRPs) identified in I *vs.* CT, PVpreI *vs.* I, PVwithI *vs.* I, PVpostI *vs.* I and PV *vs.* CT comparisons.**
