# Supplementary material for: Polyphenol dietary supplementation prevents inflammation‐induced muscle atrophy in a zebrafish ( Danio rerio ) model
Source: J Sci Food Agric. 2026 Jul 25;106(15):9581–95. doi: 10.1002/jsfa.70932 (PMC13743393; doi:10.1002/jsfa.70932)
Supplement: Supplementary file 2 [file JSFA-106-9581-s003.docx]

**Supplemental Figures**

**Figure S1.** Scheme of the TMT-label based proteomic approach used for quantitative analysis of differentially represented proteins (DRPs) in the skeletal muscle tissues of zebrafish experiencing different diet regimens. The scheme shows the different diet regimens (CT, control animals; I, animals experiencing the pro-inflammatory diet; PVpreI, animals experiencing pre-treatment with chestnut shell polyphenols and *V. macrurum* extract before the proinflammatory diet; PVwithI, animals experiencing co-treatment with chestnut shell polyphenols and *V. macrurum* extract and the proinflammatory diet; PVpostI, animals experiencing the proinflammatory diet followed by post-treatment with chestnut shell polyphenols and *V. macrurum* extract; PV, control animals fed with chestnut shell polyphenols and *V. macrurum* extract). The proteomic workflow includes sample preparation, TMT labeling, mass spectrometry analysis by nLC-ESI-Q-Orbitrap-MS/MS followed by protein identification and quantification by dedicated bioinformatics.

**Figure S2.** Volcano Plot of DRPs observed in the skeletal muscle of zebrafish experiencing different diet regimens as revealed by analysis with Proteome Discoverer software of mass spectrometric data. The Volcano plot shows the distribution of differentially represented proteins in each pairwise comparison. Significantly over- and down-represented proteins (Log_2_FC > 0.58, p < 0.05) are reported in highlighted red and green areas, respectively, while components with non-significant differences are shown in the grey region. Abbreviations related to the different diet regimens are described in Supplementary Figure S1.

**Figure S3.** STRING analysis of DRPs observed in the skeletal muscle of zebrafish experiencing different diet regimens. STRING analysis was performed with medium confidence to highlight protein interaction network of the differentially represented components ascertained in this study, providing insights into their potential biological roles and functional associations.
